# Could calcitonin gene‐related peptide inhibitors prevent status epilepticus?

**DOI:** 10.1002/epi.70436

**Published:** 2026-08-13

**Authors:** Michael Nsaka, Noor Baban, Sepideh Allahdadian, Armin Scheffler, Diana Lindner, Dagny Holle, Alain Lekoubou

**Affiliations:** ^1^ Department of Neurology Milton S. Hershey Medical Center, Penn State College of Medicine Hershey Pennsylvania USA; ^2^ Department of Neurology and Centre for Translational Neuro‐ and Behavioral Sciences (C‐TNBS), West German Headache Centre, University Hospital Essen University of Duisburg‐Essen Essen Germany; ^3^ Drexel University College of Medicine Philadelphia Pennsylvania USA; ^4^ Department of Public Health Sciences, A210 Penn State College of Medicine Hershey Pennsylvania USA

**Keywords:** CGRP inhibitors, epilepsy, migraine, neuroinflammation, status epilepticus

## Abstract

Calcitonin gene‐related peptide (CGRP) inhibitors modulate pain and neuroinflammation, a recognized contributor to epileptogenesis and status epilepticus (SE). We examined the association between CGRP inhibitor use and SE hospitalization in epilepsy patients with comorbid migraine. Using TriNetX (130 healthcare organizations), we identified patients with epilepsy from May 2018 through May 2026, and propensity‐matched cohorts for age, sex, race, anti‐seizure medications (ASMs), migraine diagnosis, migraine treatments, and cognitive impairment. The primary outcome was SE occurrence. Following matching, 17 738 patients were on CGRP inhibitors and 18 594 were not. SE occurred in 427 (2.41%) and 860 (4.63%) patients, respectively. Mean age (±SD) was 48.4 ± 14.9 vs 48.9 ± 17.0 years. Risk difference was −2.2% (95% confidence interval [CI]: −2.6% to −1.8%, *p* < .001), risk ratio 0.52 (95% CI: 0.46–0.58), and odds ratio 0.51 (95% CI: 0.45–0.57). SE‐related hospital visits were significantly fewer in the CGRP inhibitor group (*t* = −6.60, *p* < .001). CGRP inhibitor use was associated with reduced SE occurrence and hospitalization, supporting further investigation in patients with comorbid migraine and epilepsy.

## 1 INTRODUCTION

Calcitonin gene‐related peptide (CGRP) inhibitors have become an established therapy for migraine, targeting a neuropeptide central to pain signaling. Beyond migraine, CGRP has been implicated in mechanisms relevant to epilepsy, including modulation of cortical excitability and neuroinflammatory processes.^1^ Experimental models demonstrate that CGRP enhances cortical excitability, induces epileptiform activity, and promotes neuroinflammatory responses, effects that are attenuated by CGRP receptor antagonism.^2^ These observations suggest that CGRP signaling may be relevant to seizure susceptibility.

Building on this preclinical evidence, this work explores the clinical relevance of CGRP inhibition in epilepsy by examining its association with status epilepticus (SE). Using hospitalization for SE as a marker of verified ictal activity, we conducted an exploratory retrospective analysis to assess whether CGRP antibody therapy for migraine is associated with subsequent SE‐related hospitalization in patients with a prior history of epilepsy. This approach seeks to evaluate an existing neuromodulatory target within a clinically meaningful context, particularly in patients with comorbid migraine and epilepsy.

## 2 MATERIALS AND METHODS

### 2.1 Data source

All data and materials utilized in this study are accessible through TriNetX, a global health research network that offers comprehensive access to electronic medical records, including diagnoses, procedures, medications, laboratory results, and genomic data, across numerous healthcare organizations. The analysis for this report was conducted using data from the Global Collaborative Network, a network encompassing 130 healthcare organizations worldwide ensuring a comprehensive view of patient outcomes, treatment effectiveness, and healthcare trends.

### 2.2 Case identification

This analysis was conducted using the Global Collaborative Network, querying 130 healthcare organizations (HCOs) worldwide. The study compared outcomes between two cohorts: Cohort A consisted of patients with epilepsy within on/after May 17, 2018 (U.S. Food and Drug Administration [FDA] approval date for erenumab, the first CGRP‐targeting therapy), who were receiving CGRP inhibitors, whereas Cohort B included patients with epilepsy who were not receiving CGRP antagonists during the same period. Patients in Cohort A had a history of migraine, as this is currently the only indication for prescription of CGRP therapy. A total of 25 046 patients met the preliminary criteria for inclusion in Cohort A, and 1 886 041 patients in Cohort B.

Cohorts were then balanced using propensity score matching for age, sex, race, ASMs, migraine diagnosis, acute and preventive migraine treatments including non‐steroidal anti‐inflammatory drugs (NSAIDs), acetaminophen, aspirin, barbiturates, triptans, ergot alkaloids, and lasmiditan, as well as cognitive impairment to account for potential confounders. Full baseline characteristics before/after matching are presented in Table S1. A total of 17 738 patients in Cohort A and 18 594 patients in Cohort B were available for the final analysis.

### 2.3 Outcome assessment

An outcome was defined as a documented event of status epilepticus.

### 2.4 Statistical analysis

Statistical analyses were performed using the TriNetX platform. We calculated risk ratios, risk differences, odds ratios, and conducted *t*‐tests for comparative analysis. A *p*‐value <0.05 was considered the threshold for statistical significance.

## 3 RESULTS

A total of 25 046 patients with epilepsy on CGRP inhibitors and 1 886 041 patients with epilepsy not on CGRP inhibitors were identified from 130 healthcare organizations. Following propensity matching, we identified 17 738 patients with epilepsy on CGRP inhibitors and 18 594 patients with epilepsy not on CGRP inhibitors, which were followed through an 8‐year period. A total of 427 (2.41%) and 860 (4.63%) patients with epilepsy on and not on CGRP inhibitors, respectively, were found to have had a documented event of SE during the 8‐year follow‐up time (Table 1). Patients on CGRP inhibitors had a mean age (±SD) of 48.4 ± 14.9 years, whereas those not on CGRP inhibitors were had a mean age of 48.9 ± 17.0 years. Risk difference calculated between both arms (CGRP vs no CGRP) was −2.2%, *p* < .001, 95% confidence interval (CI): −2.6% to −1.8%. Risk ratio was 0.52, 95% CI: 0.46–0.58, and odds ratio (OR) of 0.51, 95% CI: 0.45–0.57. Number Needed to Treat (NNT): 45 with 95% CI: 39–56. Hospital visits due to SE were significantly less, *t* = −6.60, *df* = 36 330, *p* < .001 in patients with epilepsy on CGRP inhibitors compared to patients with epilepsy not on CGRP inhibitors.

**TABLE 1:**
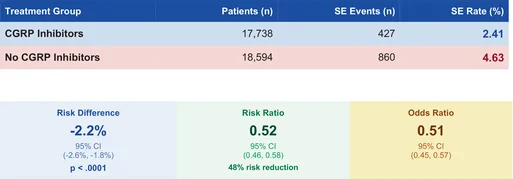
Status epilepticus (SE) incidence in propensity‐matched (see Table S1) epilepsy patients with and without CGRP inhibitor therapy over an 8‐year follow‐up period.

*Note*: Primary analysis demonstrates significant reduction in SE occurrence among patients on CGRP inhibitors compared to those not on CGRP inhibitors (2.41% vs 4.63%; RR 0.52, 95% CI: 0.46–0.58, *p* < .001), representing a 48% relative risk reduction.

Abbreviations: CGRP, calcitonin gene‐related peptide; CI, confidence interval; SE, status epilepticus.

## 4 DISCUSSION

SE remains a major problem among patients diagnosed with epilepsy. Lifetime occurrence has been estimated at ~63% in infancy, 47% between 2 and 9 years of age, and 21%–31% thereafter.^3^ Despite advances in ASMs targeting ion channels and synaptic transmission, disease‐modifying strategies for SE are limited.^4^

SE is characterized by sustained neuronal hyperexcitability resulting from failure of inhibitory networks in the context of overwhelming excitotoxicity.^4^ Neuroinflammation has emerged as a central contributor to this process, promoting blood–brain barrier disruption and perpetuating seizure activity.^5^, ^6^ CGRP is a key neuropeptide implicated in both excitatory signaling and neuroinflammatory pathways.^1^, ^2^ Preclinical evidence supports its role in epileptiform activity, with antagonism of CGRP with olcegepant reducing cortical spreading depression amplitude, propagation, and epileptiform discharges in vivo, whereas CGRP administration induces synchronized cortical bursting in mouse models.^1^, ^2^, ^7^ These findings support a pathophysiological role for CGRP in promoting cortical hyperexcitability. Further convergent evidence comes from studies of perampanel, an AMPA receptor antagonist, which inhibits basal and stimulated CGRP release from isolated rat brainstem tissue in a concentration‐dependent manner.^8^ This effect was observed with potassium chloride– and veratridine‐induced CGRP release but not with capsaicin‐induced release,^8^ with potential explanations including differential CGRP peptidoforms or capsaicin‐induced release exceeding available inhibitory concentrations. Together, these data suggest convergence between glutamatergic transmission and CGRP‐mediated signaling in regulating neuronal excitability and provide biological plausibility for CGRP modulation as a therapeutic target in epilepsy. Based on these observations, we hypothesized that CGRP inhibition enhances dysfunctional inhibitory pathways in patients with epilepsy at risk for SE. Our findings support this hypothesis and suggest that CGRP inhibition may influence mechanisms relevant to seizure propagation and persistence. Although causal inference cannot be established, the observed association aligns with experimental data linking CGRP to excitability and neuroinflammation.^2^, ^5^, ^7^, ^8^, ^9^

Confounding by indication is a limitation in our work because migraine patients prescribed CGRP inhibitors likely differ systematically from those who are not, and because migraine itself shares pathophysiology with epilepsy, residual confounding cannot be fully excluded despite propensity matching, particularly given our observational study design. With that caveat, migraine (for which CGRP inhibitors are currently approved) shares overlapping pathophysiological mechanisms with epilepsy, including cortical spreading depression and neuroinflammatory signaling.^10^, ^11^ CGRP inhibition may therefore offer dual benefit in patients with comorbid migraine and epilepsy, potentially reducing both migraine burden and severe SE‐related complications such as hyperthermia, prolonged encephalopathy, and focal neurological deficits.^6^ CGRP inhibition may also have implications for migralepsy, a rare condition in which seizures are triggered by or follow migraine with aura.^12^, ^13^ Shared mechanisms between migraine and epilepsy support the biological plausibility of CGRP inhibition as a preventive strategy in this population, which may be underserved by antiseizure therapy alone.^12^, ^14^, ^15^ The potential relevance in pediatric epilepsy is also a consideration, although CGRP‐based therapies are not currently approved for individuals younger than 18 years of age, and focused investigation in pediatric populations is warranted. Other limitations of our work include its retrospective observational nature and residual confounding risk. Migraine severity, epilepsy‐related severity, functional status, cognitive impairment, communication ability, and activities of daily living were not available and therefore not accounted for. Furthermore, CGRP‐targeting therapies are approved and reimbursed primarily for migraine and access is disproportionately influenced by insurance coverage and socioeconomic factors, representing an additional unmeasured source of confounding by indication that propensity matching cannot fully address and may limit the internal validity of the findings.^12^

## 5 CONCLUSION

In this large database, using propensity matching, CGRP inhibitors were associated with a 48% lower rate of SE hospitalization among patients with epilepsy. This is, however, an approximate descriptive estimate from observational data and should not be interpreted as causal. Given the known overlap between CGRP‐mediated pathways and neuronal excitability, this association may be hypothesis generating for shared mechanisms between migraine and seizure susceptibility. Prospective studies, including randomized clinical trials, are needed to establish causality and clinical utility.

## AUTHOR CONTRIBUTIONS

M.N. and A.L. designed and conceptualized the study, interpreted the data, and drafted the manuscript. M.N. was responsible for statistical analysis. S.A., N.B., A.S., D.L., and D.H. interpreted the data and revised the manuscript for intellectual content. All authors read and approved the final manuscript.

## FUNDING INFORMATION

No funding was received for this work.

## CONFLICT OF INTEREST STATEMENT

M.N. received travel fees from Licher MT. D.H. has received scientific support and/or honoraria from Biogen, Novartis, Eli Lilly, Sanofi Aventis, Teva, Allergan, and Hormosan. D.L. received honoraria from Novartis. A.S. has received travel fees and/or honoraria from Teva and Novartis. The remaining authors have no conflicts of interest.

## ETHICS STATEMENT

We confirm that we have read the Journal's position on issues involved in ethical publication and affirm that this report is consistent with those guidelines.

## Supporting information


**Table S1.** Baseline characteristics before and after propensity score matching.

## Data Availability

The data that support the findings of this study are available from TriNetX (https://www.trinetx.com), specifically the TriNetX Global Collaborative Network. Restrictions apply to the availability of these data, which were accessed and analyzed under a Data Use Agreement between the authors' institution and TriNetX, and are therefore not publicly available. Researchers seeking access to comparable de‐identified data may do so by establishing their own agreement with TriNetX, subject to TriNetX's approval.
