# Supplementary material for: Could calcitonin gene‐related peptide inhibitors prevent status epilepticus?
Source: Epilepsia. 2026 Aug 13;67(10):e198–201. doi: 10.1002/epi.70436 (PMC13675781; doi:10.1002/epi.70436)
Supplement: Supplementary file 1 [file EPI-67-e198-s001.pdf]

Baseline Characteristics Before and After Propensity Score Matching						
	Before Matching			After Matching		
Characteristic	CGRP Group (N = 25,046) % or Mean ± SD	Control Group (N = 1,886,041) % or Mean ± SD	SMD	CGRP Group (N = 22,379) % or Mean ± SD	Control Group (N = 22,379) % or Mean ± SD	SMD
**Demographics**						
Age, mean ± SD (years)	48.1 ± 14.8	47.9 ± 25.0	0.005	48.4 ± 14.9	48.9 ± 17.0	0.037
Female, n (%)	20,711 (82.7%)	890,933 (49.4%)	0.752	18,221 (81.4%)	18,655 (83.4%)	0.051
Male, n (%)	4,315 (17.2%)	912,072 (50.5%)	0.752	4,138 (18.5%)	3,703 (16.5%)	0.051
**Race**						
White, n (%)	19,012 (75.9%)	990,165 (54.9%)	0.453	16,778 (75.0%)	17,121 (76.5%)	0.036
Black or African American, n (%)	3,163 (12.6%)	279,745 (15.5%)	0.083	2,928 (13.1%)	3,002 (13.4%)	0.010
Hispanic or Latino, n (%)	2,004 (8.0%)	147,156 (8.2%)	0.006	1,809 (8.1%)	1,756 (7.8%)	0.009
Asian, n (%)	272 (1.1%)	62,383 (3.5%)	0.160	257 (1.1%)	209 (0.9%)	0.021
Other Race, n (%)	759 (3.0%)	81,752 (4.5%)	0.079	700 (3.1%)	590 (2.6%)	0.029
American Indian or Alaska Native, n (%)	160 (0.6%)	9,883 (0.5%)	0.012	146 (0.7%)	148 (0.7%)	0.001
Native Hawaiian or Other Pacific Islander, n (%)	85 (0.3%)	9,351 (0.5%)	0.027	74 (0.3%)	77 (0.3%)	0.002
Unknown Race, n (%)	1,595 (6.4%)	371,265 (20.6%)	0.425	1,496 (6.7%)	1,232 (5.5%)	0.049
**Ethnicity**						
Not Hispanic or Latino, n (%)	19,830 (79.2%)	1,095,860 (60.7%)	0.411	17,561 (78.5%)	17,760 (79.4%)	0.022
Unknown Ethnicity, n (%)	3,212 (12.8%)	561,528 (31.1%)	0.453	3,009 (13.4%)	2,863 (12.8%)	0.019
**Comorbid Diagnoses**						
Migraine (G43), n (%)	19,155 (76.5%)	64,846 (3.6%)	2.225	16,488 (73.7%)	16,988 (75.9%)	0.051
Other specified cognitive deficit (R41.84), n (%)	943 (3.8%)	11,898 (0.7%)	0.212	770 (3.4%)	750 (3.4%)	0.005
**Medications**						

Anticonvulsants, n (%)	21,594 (86.2%)	502,728 (27.9%)	1.459	18,932 (84.6%)	18,923 (84.6%)	0.001
Opioid analgesics, n (%)	18,519 (73.9%)	474,694 (26.3%)	1.084	16,112 (72.0%)	16,471 (73.6%)	0.036
Acetaminophen, n (%)	19,127 (76.4%)	535,412 (29.7%)	1.059	16,672 (74.5%)	16,854 (75.3%)	0.019
NSAIDs, n (%)	11,448 (45.7%)	232,179 (12.9%)	0.774	9,742 (43.5%)	9,827 (43.9%)	0.008
Beta-blocking agents, n (%)	12,369 (49.4%)	266,922 (14.8%)	0.798	10,497 (46.9%)	10,390 (46.4%)	0.010
Topiramate, n (%)	11,282 (45.0%)	41,612 (2.3%)	1.163	9,060 (40.5%)	8,923 (39.9%)	0.012
Sumatriptan, n (%)	8,855 (35.4%)	17,223 (1.0%)	0.997	6,812 (30.4%)	6,504 (29.1%)	0.030
Aspirin, n (%)	7,959 (31.8%)	200,825 (11.1%)	0.520	6,911 (30.9%)	6,875 (30.7%)	0.003
Opioid antagonist analgesics, n (%)	6,545 (26.1%)	98,471 (5.5%)	0.591	5,508 (24.6%)	5,517 (24.7%)	0.001
Valproate, n (%)	5,078 (20.3%)	81,885 (4.5%)	0.492	4,041 (18.1%)	3,847 (17.2%)	0.023
Rizatriptan, n (%)	5,327 (21.3%)	5,530 (0.3%)	0.718	3,497 (15.6%)	2,893 (12.9%)	0.077
Butalbital, n (%)	5,642 (22.5%)	25,934 (1.4%)	0.687	4,512 (20.2%)	4,588 (20.5%)	0.008
Naratriptan, n (%)	1,309 (5.2%)	649 (0.0%)	0.329	628 (2.8%)	409 (1.8%)	0.065
Eletriptan, n (%)	1,242 (5.0%)	1,235 (0.1%)	0.316	788 (3.5%)	639 (2.9%)	0.038
Zolmitriptan, n (%)	1,094 (4.4%)	1,030 (0.1%)	0.296	651 (2.9%)	510 (2.3%)	0.040
Dihydroergotamine, n (%)	829 (3.3%)	1,049 (0.1%)	0.255	497 (2.2%)	360 (1.6%)	0.045
Methylergonovine, n (%)	546 (2.2%)	5,266 (0.3%)	0.172	449 (2.0%)	477 (2.1%)	0.009
Frovatriptan, n (%)	332 (1.3%)	237 (0.0%)	0.161	198 (0.9%)	141 (0.6%)	0.029
Lasmiditan, n (%)	185 (0.7%)	29 (0.0%)	0.122	58 (0.3%)	23 (0.1%)	0.037
Almotriptan, n (%)	176 (0.7%)	231 (0.0%)	0.116	113 (0.5%)	93 (0.4%)	0.013
Ergotamine, n (%)	86 (0.3%)	549 (0.0%)	0.073	58 (0.3%)	55 (0.2%)	0.003

SMD = Standardized Mean Difference. SMD < 0.10 indicates adequate balance. Propensity score matching was performed using the TriNetX Global Collaborative Network platform (130 healthcare organizations) on a 1:1 basis. All covariates listed above were included in the propensity score model. NSAIDs = Non-steroidal anti-inflammatory drugs.

Propensity score matching was performed using the TriNetX Global Collaborative Network platform. Propensity scores were estimated via logistic regression incorporating all listed covariates. Matching was conducted using a 1:1 nearest-neighbor greedy algorithm without replacement, with a caliper width of 0.10 standard deviations of the propensity score. Cohorts were randomly shuffled prior to matching. Handling of missing covariate data follows TriNetX platform defaults, which are not explicitly documented; this represents a limitation of the platform-based analysis.

***Supplementary Table** showing Baseline Characteristics Before and After Propensity Score Matching*
